# Meconium Indicators of Maternal Alcohol Abuse during Pregnancy and Association with Patient Characteristics

**DOI:** 10.1155/2014/702848

**Published:** 2014-03-30

**Authors:** Tamme W. Goecke, Pascal Burger, Peter A. Fasching, Abdulsallam Bakdash, Anne Engel, Lothar Häberle, Franziska Voigt, Florian Faschingbauer, Eva Raabe, Nicolai Maass, Michael Rothe, Matthias W. Beckmann, Fritz Pragst, Johannes Kornhuber

**Affiliations:** ^1^Department of Obstetrics and Gynecology, Friedrich-Alexander-University of Erlangen-Nuremberg, Universitaetsstraß 21-23, 91054 Erlangen, Germany; ^2^Department of Obstetrics and Gynecology, University of RWTH Aachen, Pauwelsstrß 30, 52074 Aachen, Germany; ^3^Department of Psychiatry and Psychotherapy, Friedrich-Alexander-University of Erlangen-Nuremberg, Schwabachanlage 6-10, 91054 Erlangen, Germany; ^4^Institute of Legal Medicine, University Hospital Charité, Hittorfstraße 18, 14195 Berlin, Germany; ^5^Lipidomix GmbH, Berliner Allee 261-269, 13088 Berlin, Germany

## Abstract

*Aim*. Identification of women with moderate alcohol abuse during pregnancy is difficult. We correlated self-reported alcohol consumption during pregnancy and patient characteristics with objective alcohol indicators measured in fetal meconium. *Methods*. A total of 557 women singleton births and available psychological tests, obstetric data and meconium samples were included in statistical analysis. Alcohol metabolites (fatty acid ethyl esters (FAEEs) and ethyl glucuronide (EtG)), were determined from meconium and correlated with patient characteristics. *Results*. We found that 21.2% of the 557 participants admitted low-to-moderate alcohol consumption during pregnancy. Of the parameters analyzed from meconium, only EtG showed an association with alcohol history (*P* < 0.01). This association was inverse in cases with EtG value above 120 ng/g. These values indicate women with most severe alcohol consumption, who obviously denied having consumed alcohol during pregnancy. No other associations between socioeconomic or psychological characteristics and the drinking status (via meconium alcohol metabolites) could be found. *Conclusion*. Women who drink higher doses of ethanol during pregnancy, according to metabolite measures in meconium, might be less likely to admit alcohol consumption. No profile of socioeconomic or psychological characteristics of those women positively tested via meconium could be established.

## 1. Introduction

Self-reported maternal alcohol abuse during pregnancy is not reliable and ethanol consumption is rarely admitted, if at all [1, 2]. However, alcohol consumption during pregnancy is a relevant problem, with its estimated prevalence ranging from 3.5 up to 53.9% in European countries [1–3]. Excessive prenatal alcohol exposure is reported to be associated with severe consequences for the fetus, such as premature birth, miscarriages, fetal alcohol syndrome (FAS) or fetal alcohol spectrum disease (FASD), and other physical and neuropsychological disorders [4–6].

Even low or moderate alcohol exposure may lead to a higher perinatal mortality [7], and it may cause congenital anomalies. In addition, the risk increases with higher dose [8, 9]. The role of moderate alcohol consumption in pregnant women is controversially discussed [3, 10–15], particularly because an exact dose-effect correlation between alcohol intake and development of physical and neuropsychological problems could not be established. Therefore, a labeling for alcoholic drinks and a recommendation of complete abstinence during pregnancy have been established in many European countries, such as France.

Commonly performed laboratory tests for alcohol consumption such as carbohydrate deficient transferrin (CDT), liver enzymes such as gamma-glutamyl-transferase (GGT), and mean corpuscular volume (MCV) are indirect alcohol markers. They are difficult to interpret and insufficiently reliable during pregnancy [16, 17].

Other parameters for alcohol consumption exist as direct metabolites of ethanol degradation. They can be found in blood, urine, hair, and meconium. Fatty acid ethyl esters (FAEE) in meconium have been investigated and established in several studies as biomarkers of fetal ethanol exposure during the last 3 months of pregnancy [1, 18–20]. Additionally, the determination of ethylglucuronide (EtG) not only from the mothers' hair or urine but also from the children's meconium has been associated with the mother's drinking behavior during pregnancy [2, 20–23].

In our study, we aimed at the assessment of the association between patients' self-reported alcohol intake and meconium biomarkers for maternal alcohol consumption.

Furthermore, we tried to identify the characteristics of mothers, who, according to direct ethanol metabolites, presumably drank during their pregnancy, using epidemiological and medical history, and we standardized the psychological questionnaires.

## 2. Materials and Methods

The Franconian Maternal Health Evaluation Studies (FRAMES) were prospectively conducted from 2005 to 2007 [24–27]. A total of 1100 women were recruited as a consecutive cohort. The participating women had to be aged ≥ 18 years with at least 30 full weeks of gestational age, and were introduced as outpatients to the Department of Obstetrics and Gynecology of the University of Erlangen-Nuremberg. There was no preselection of the cases with respect to suspected alcohol abuse of the mother or any other parameter.

Only singleton births were allowed for this analysis, resulting in the exclusion of 114 births. Therefore, the final number of participants in this study was 986. Further, 247 newborns were transferred to the children's hospital because of perinatal problems and were excluded as well. Of the left 739 newborns, 137 had to be excluded because of missing consent of the mother or sampling was missed. From the remaining 602 samples, 45 could not be investigated due to technical issues (i.e., too little sample volume). There were no statistical differences between women with available meconium measurements (557) and those without.

This study was approved by the Local Ethics Committee of the Medical Faculty of the University of Erlangen-Nuremberg and was conducted in accordance with the Declaration of Helsinki and all patients gave informed consent.

All participating women were interviewed with standardized psychological questionnaires for the identification of comorbid psychiatric disorders at three occasions. The first was done prenatally from the 30th week of pregnancy onward (first contact with the pregnant women), the second was done 48–72 hours postpartum (when the meconium was collected), and the third was carried out 6–8 months postpartum. Furthermore, we collected diagnostic, obstetric, and medical history from the women.

Psychological questionnaires for other psychiatric disorders were comprised, including the Hamilton rating scale for depression (HAMD), the Edinburgh Postnatal Depression Scale (EPDS), and others—the results of these evaluations are published elsewhere. In addition, anamnestic data on partnership, sexual life, and social status as well as medical parameters of the mother and child were acquired.

About 1 g of meconium was collected from the newborns within the first 2–24 h after birth and frozen at −80°C for up to 30 months until analysis. The meconium samples were analyzed in the Department of Forensic Toxicology of the Institute of Legal Medicine, University Hospital Charité, and by the Lipidomix GmbH in Berlin. The procedure for determination of FAEE and EtG has been described in detail in a previous paper [20]. The FAEEs ethyl Myristate (E14), ethyl Palmitate (E16), ethyl Linoleate (E18:2), ethyl Oleate (E18:1), and ethyl Stearate (E18), as well as the corresponding deuterated standards D5-FAEE, were purchased or prepared as described previously [20, 28]. The quantification of FAEE in meconium was performed according to an optimized and validated method described previously [23].

EtG was determined in meconium according to a liquid-chromatography/tandem-mass-spectrometry (LC-MS-MS) procedure with D5-EtG as the internal standard in analogy to the measurement in hair [21]. The measurement was performed by LC-MS-MS as described in our previous paper [20].

### 2.1. Statistical Considerations

Univariant associations of alcohol history, meconium results, and socioeconomic parameters were analyzed with appropriate statistical tests. Wilcoxon rank-sum tests were used for ordinal parameters, and *χ* ^2^ tests were used for categorical (i.e., yes/no) parameters. All measures of meconium concentrations are reported in ng per g meconium.

**Figure 1:**
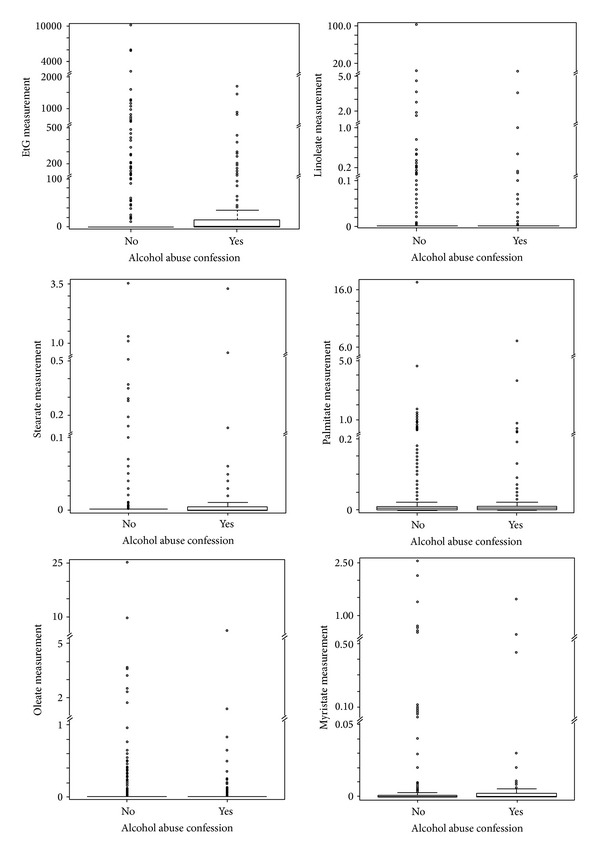
Boxplots of EtG and ester measures and alcohol abuse confession. Suitable ranges are displayed.

In a preanalysis, boxplots were generated to get a first impression of the meconium data distributions in the two groups of reported alcohol consumption: yes (Y) and no (N). The distributions were asymmetric with many outliers in the group with no reported alcohol consumption as well as higher values for the majority of measurements in the group with reported alcohol consumption (Figure 1). We therefore hypothesized a cutoff point, which could divide the women into two groups: one with at least moderate measurements, which would have a positive association of the meconium results with alcohol history, and a second with higher meconium results, in which this association would be negative. To find an optimal cutoff, thresholds were run between the 10th and the 90th percentile of all meconium measurements. For each choice of the threshold, both the subgroups (Group 1 < cutoff, Group 2 ≥ cutoff) were separately tested to determine the differences between the abusers and nonabusers with Wilcoxon rank-sum tests. The optimal cutoff point was defined as the minimum sum of *P* values from both tests.

Multiple logistic regression models with meconium measures (=0 versus >0, resp., <cutoff point versus ≥cutoff point) as target variables and socioeconomic parameters as predictive variables were performed to calculate the odds ratios (OR). The final models were obtained by the backward stepwise variable selection due to the Akaike information criterion. For each model, the area under the curve (AUC) of the receiver operation characteristic (ROC), ranging from 0.5 (random prediction) to 1 (perfect prediction), was calculated to summarize the strength of prediction.

All tests were two-sided, and a *P* value of <0.05 was considered to be statistically significant. The analyses were carried out using the R system for statistical computing (version 2.11.1; R Development Core Team, Vienna, Austria, 2010).

## 3. Results

### 3.1. Main Patient Characteristics

The final number of participating women with singleton birth in this study was 986. In average, participants were 32 years old and 557 gave birth to their first child in this study. Average pregnancy at the time of birth was 1.9; 514 were boys and 470 were girls. The average weight for the boys was 3497 g and the average weight for the girls was 3318 g.

A total of 204 women (20.8%) confessed to have drunk alcohol at some time point to some extent during pregnancy. Most women stated to have drunk moderately alcohol with a low frequency. None of the women admitted to having drunk alcohol extensively.

### 3.2. Main Measurement Characteristics

With regard to the meconium measures (Linoleate, Palmitate, Stearate, Oleate, Myristate, and EtG), most of the samples were free of fatty esters and EtG. The percentage of negative samples reached from 22% to 84% in the group of the fatty esters and was 83% for EtG. The maximum values were rather low for Myristate and Stearate with 2.6 and 3.6, respectively, and reached values of 103.2 with regard to Linoleate. Oleate and Palmitate maximum values were in between with 25.4 and 17.4. Maximum value for EtG was 10,235. The distribution of measurements is shown in Figure 1.

### 3.3. Association of Alcohol Abuse Confession with Meconium Measures

For the majority of meconium measures, the strong outliers seen in the boxplots of the meconium measures result in a higher mean in the group of alcohol abuse confessing women compared to the group of women who did not confess alcohol abuse. The mean EtG value in the group of alcohol abuse confessing women was 71 and it was 110 in the group of women who did not confess alcohol abuse. The corresponding values for Linoleate were 0.30 versus 0.05, for Oleate were 0.16 versus 0.11, and for Myristate were 0.023 versus 0.018.

Rank based testing confirmed only for EtG the observation that the group with alcohol abuse confession showed overall higher measurements (*P* < 0.01, Wilcoxon rank-sum test) (Table 1). Additionally, the five meconium measures Linoleate, Palmitate, Stearate, Oleate, and Myristate were analyzed with other statistical methods without any significant results (data not shown).

Only for EtG, an optimal cutoff with significant *P* values was found. In fact, this cutoff was at 120 ng/g for EtG. When looking only at the group of women with an EtG < 120ng/g, those women denying alcohol abuse had mean EtG values of 3.0 ng/mL, while those women admitting alcohol abuse had mean EtG values of 6.6 ng/mL (*P* < 0.001). Above the cutoff, the mean EtG value was 482.2 for women with alcohol abuse confession and 1179.8 for women without alcohol abuse confession (*P* = 0.055). For all other meconium measures, the optimal cut point had no significant *P* values (data not shown). Due to small sample size, this cutoff could not be validated.

Based on these results, further analyses with the alcohol abuse confession as target variable were conducted separately for the two subgroups defined by the EtG cutoff point.

### 3.4. Association of Meconium Measures with Socioeconomic Parameters

In order to understand whether there is a correlation between commonly known socioeconomic factors which might be correlated with alcohol abuse, both, the alcohol abuse confession variable and the meconium measures, were correlated with socioeconomic characteristics. Except for an association between smoking status and Oleate measures and marital status and Myristate measures, no associations were seen. Women who were smokers had more often an elevated Oleate measure (38.1% versus 23.2%; *P* = 0.049), and women who were married had less frequently elevated Myristate measures (30.2% versus 45.9%; *P* < 0.01). None of these tests were adjusted for multiple testing though, and none of the findings were consistent with other fatty ester measures or EtG. The associations between EtG and socioeconomic parameters for EtG with the determined cutoff of 120 ng/g are shown in Table 2.

### 3.5. Association between Alcohol Abuse Confession and Socioeconomic Factors

Comparing the women's statements about alcohol abuse confession revealed only an association between alcohol abuse and age of women with an EtG measure above 120 ng/g (Tables 3 and 4).

### 3.6. Multivariate Models

Thinking of objective alcohol meconium measures as variables which might be helpful in clinical practice to predict, socioeconomic factors and alcohol abuse confession as independent variables, and meconium measures as dependent variables (six unique models) were analyzed with logistic regression models. Nontrivial final models (i.e., with at least one independent variable) are shown in Table 5. The selection procedure does not leave any independent variables in models for alcohol and Stearate.

## 4. Discussion

In this prospective study, we found EtG to be associated with self-reported alcohol abuse in women with low-to-moderate alcohol consumption. However, in women, whose meconium EtG indicated a severe alcohol abuse, there was an inverse correlation with self-reported alcohol abuse, indicating that women with severe alcohol abuse might most likely be the ones to deny the alcohol abuse.

Our results might be a hint that women who drink more heavily during their pregnancy are not likely to admit their drinking status truthfully compared to those who moderately consume alcohol.

The cutoff values for objective alcohol parameters, which are currently found and tested in science and research, are not yet established due to several reasons. Nutritional and other environmental factors can influence the amount of nonoxidative alcohol metabolites [29]. There are small amounts of FAEE in meconium of neonates without active maternal alcohol consumption, which may originate from endogenous ethanol or from ethanol traces contained in common foods [30–32]. In contrast to the analysis of EtG and FAEEs in the patients' hair, there are no established and scientifically tested cutoff values for differentiating the mothers' drinking behavior via meconium, and imagining a reliable, scientifically correct, and ethical way to test the cutoff values in pregnant women is hardly possible [33]. Taking this into consideration, a strict differentiation between teetotaler, low-to-moderate, and high-risk drinkers cannot be realized. The absolute values of FAEEs and EtG in meconium can only provide a hint about the degree of the mother's ethanol consumption. Using meconium as the material for analysis, we can overlook the last trimenon of pregnancy, as meconium is accumulated in the fetal gut from about the 20th week of gestation until birth, and the major amount of it is observed during the last weeks before birth. Therefore, at least 75% of the sample material originates from the last 8 weeks of pregnancy [34, 35].

Although we had a rather large sample with 986 participants and 557 analyzed meconium samples, the positive cases—according to the toxicological meconium measures—were only a small percentage of the participants. Consecutively, the statistical power was also limited and no cross-validation or adjustment for multiple testing was possible.

In other studies, FAEEs and EtG were already used as parameters to identify alcohol drinking mothers, and they showed partly drastic differences between the admitted drinking behavior and the one shown by using objective parameters [20–22, 36–38]. Our data are based on a large sample, and we use not only the mothers‘ statements, but also psychological questionnaires and objective alcohol parameters alongside each other.

Questionnaires and laboratory blood parameters used in the common routine of alcohol diagnostics are not reliable sources of information in pregnant women [2, 16, 17]. An objective evaluation of drinking status can be achieved using direct alcohol metabolites, such as EtG and FAEEs [18, 21, 28, 39], which have been shown to be parameters for the mothers‘ alcohol consumption [2, 19, 20, 40, 41].

Although there is no established cutoff value, Moore et al. [40] concluded that a total cumulative FAEE concentration of >10,000 ng/g may indicate that the newborn has been exposed to significant amounts of alcohol during gestation. Three of the meconium samples in our study showed higher values, with two of those women completely denying alcohol abuse during pregnancy.

Most of the women denied alcohol consumption and most had completely negative results concerning EtG; moreover, the mean value and rank-based tests showed higher values in the group of women admitting alcohol consumption during pregnancy. These findings seem logical if we act on the assumption that the women answered the question of drinking ethanol truthfully. Still, we found the characteristic cutoff to be at 120 ng/g of EtG, under which the women‘s admitting status correlated positively with the meconium measures, and above which the correlation was inverse. This paradox phenomenon invigorates the findings of many other studies that suggest an unreliability of maternal statements concerning alcohol consumption during pregnancy [1, 2, 33].

## 5. Conclusion

There is an ongoing immense need for further investigations in the field of alcohol consumption during pregnancy, because in Germany alone, every year, about 4000 newborn children suffer from the FAS, and even more are those born with symptoms of the fetal alcohol spectrum disease. After validation, alcohol screening could be implemented systematically in prenatal care [42, 43] as well as the postnatal identification of women being at high risk for child neglect [44, 45].

We found that women with a high alcohol consumption are more likely to deny their alcohol abuse. This finding is of clinical and scientific importance. Identifying women with a severe alcohol problem as the ones who would most likely not admit their problem might indicate an even higher risk for the unborn child, as those pregnancies are more difficult to identify. Additionally, prediction models using self-reported alcohol abuse might be more complicated as there is a positive correlation with truth in women with low-to-moderate consumption and an inverse correlation with truth in women with severe alcohol abuse. More studies are needed especially confirming meconium measurements with clinical parameters concerning fetal and pediatric outcome to test their reliability concerning clinical and scientific use.

## Figures and Tables

**Table 1:** Association of meconium results, report of alcohol consumption count and percentage, and *P* values of Wilcoxon rank-sum tests are shown (test based on raw values, classifications just for illustration).

Meconium results	ng/g	Total	No alcohol abuse reported	Alcohol abuse reported	*P* value
Stearate	0	411	80.8%	19.2%	0.08
	>0	139	72.7%	27.3%	

Linoleate	0	460	79.1%	20.9%	0.66
	>0	90	76.7%	23.3%	

Oleate	0	416	78.8%	21.2%	0.75
	>0	134	78.4%	21.6%	

Palmitate	0	121	80.2%	19.8%	0.55
	>0	423	78.5%	21.5%	

Myristate	0	367	79.3%	20.7%	0.76
	>0	183	77.6%	22.4%	

EtG	0	451	81.4%	18.6%	<0.01
	>0	92	66.3%	33.7%	

**Table 2:** Association of socioeconomic parameters and EtG measures. Count, percentage, and *P* value of nonparametric test are shown.

Patient characteristic	Total	EtG measurement <120 ng/g	EtG measurement ≥120 ng/g	*P* value
Age				
<20	3	66.7%	33.3%	
20–30	128	90.6%	9.4%	
30–40	382	90.1%	9.9%	
40+	32	90.6%	9.4%	
Total	**545**			0.92
Education				
No university-entrance diploma	245	91.8%	8.2%	
University-entrance diploma	296	88.5%	11.5%	
Total	**541**			0.25
Marital status				
Not married	110	84.5%	15.5%	
Married	434	91.5%	8.5%	
Total	**544**			0.05
Income				
<500€	1	0.0%	0.0%	
500–1000€	19	100.0%	0.0%	
1000–2000€	59	89.8%	10.2%	
2000–3000€	122	85.2%	14.8%	
3000–4000€	94	94.7%	5.3%	
4000–5000€	56	89.3%	10.7%	
>5000€	48	93.8%	6.3%	
Total	**399**			0.32
Smoking				
No	503	90.1%	9.9%	
Yes	40	90.0%	10.0%	
Total	**543**			1.00
EPDS				
No depression	428	90.0%	10.0%	
Slight depression	37	89.2%	10.8%	
Moderate-to-strong depression	36	91.7%	8.3%	
Total	**501**			0.88
HAMD				
No depression	466	91.8%	8.2%	
Slight-to-moderate depression	56	91.1%	8.9%	
Strong depression	11	90.9%	9.1%	
Total	**533**			0.77

**Table 3:** Association of socioeconomic parameters and alcohol for Group 1 (EtG < cutoff of 120 ng/g). Count, percentage, and *P* value of nonparametric test are shown.

Patient characteristics	Total	Alcohol consumption not reported in Group 1	Alcohol consumption reported in Group 1	*P* value
Age				
<20	2	100.0%	0.0%	
20–30	116	81.9%	18.1%	
30–40	342	78.4%	21.6%	
40+	29	82.8%	17.2%	
Total	**489**			0.15
Education				
No university-entrance diploma	223	78.0%	22.0%	
University-entrance diploma	262	80.9%	19.1%	
Total	**485**			0.50
Marital status				
Not married	93	78.5%	21.5%	
Married	395	79.7%	20.3%	
Total	**488**			0.90
Income				
<500€	0	0.0%	0.0%	
500–1000€	19	84.2%	15.8%	
1000–2000€	53	83.0%	17.0%	
2000–3000€	104	83.7%	16.3%	
3000–4000€	89	79.8%	20.2%	
4000–5000€	50	78.0%	22.0%	
>5000€	45	77.8%	22.2%	
Total	**360**			0.27
Smoking				
No	453	79.0%	21.0%	
Yes	36	86.1%	13.9%	
Total	**489**			0.42
EPDS				
No depression	385	78.2%	21.8%	
Slight depression	33	84.8%	15.2%	
Moderate-to-strong depression	33	78.8%	21.2%	
Total	**451**			0.54
HAMD				
No depression	428	79.2%	20.8%	
Slight-to-moderate depression	51	80.4%	19.6%	
Strong depression	10	90.0%	10.0%	
Total	**489**			0.59

**Table 4:** Association of socioeconomic parameters and reported alcohol abuse for Group 2 (EtG ≥ cutoff of 120 ng/g). Count, percentage, and *P* value of nonparametric test are shown.

Patient characteristic	Total	Alcohol consumption not reported in Group 2	Alcohol consumption reported in Group 2	*P* value
Age				
<20	1	100.0%	0.0%	
20–30	12	91.7%	8.3%	
30–40	38	68.4%	31.6%	
40+	3	33.3%	66.7%	
Total	**54**			0.03
Education				
No university-entrance diploma	20	80.0%	20.0%	
University-entrance diploma	34	67.6%	32.4%	
Total	**54**			0.51
Marital status				
Not married	17	82.4%	17.6%	
Married	37	67.6%	32.4%	
Total	**54**			0.34
Income				
<500€	1	100.0%	0.0%	
500–1000€	0	0.0%	0.0%	
1000–2000€	6	66.7%	33.3%	
2000–3000€	18	72.2%	27.8%	
3000–4000€	5	80.0%	20.0%	
4000–5000€	6	66.7%	33.3%	
>5000€	3	66.7%	33.3%	
Total	**39**			0.89
Smoking				
No	51	72.5%	27.5%	
Yes	3	66.7%	33.3%	
Total	**54**			0.31
EPDS				
No depression	43	67.4%	32.6%	
Slight depression	4	75.0%	25.0%	
Moderate-to-strong depression	3	100.0%	0.0%	
Total	**50**			0.32
HAMD				
No depression	48	72.9%	27.1%	
Slight-to-moderate depression	5	60.0%	40.0%	
Strong depression	1	100.0%	0.0%	
Total	**54**			0.79

**Table 5:** Various multiple logistic regression analyses (final models). The area under the curve (AUC) and odds ratios with 95% confidence interval and corresponding *P* values are shown.

Target variable	AUC	Predictive variables	Odds ratio	95% confidence interval	*P* value
Linoleate (0/>0)	0.58	Education			
		No university-entrance diploma	1		
		University-entrance diploma	1.74	[0.93, 3.31]	0.09
		Income			
		<500€	1		
		Per category	0.84	[0.67, 1.05]	0.13

Oleate (0/>0)	0.53	Smoking			
		No	1		
		Yes	2.05	[0.91, 4.42]	0.07

Palmitate (0/>0)	0.54	Marital status			
		Not married	1		
		Married	1.57	[0.86, 2.79]	0.13

Myristate (0/>0)	0.58	Age			
		<25	1		
		≥25	3.15	[1.06, 11.74]	0.05
		Marital status			
		Not married	1		
		Married	0.62	[0.35, 1.09]	0.09
		Income			
		<500€	1		
		Per category	0.85	[0.72, 1.01]	0.07

EtG (<120/≥120)	0.59	Education			
		No university-entrance diploma	1		
		University-entrance diploma	1.81	[0.86, 3.92]	0.12
		Income			
		<500€	1		
		Per category	0.79	[0.60, 1.04]	0.10
